# Use of endogenous signal sequences for transient production and efficient secretion by moss (*Physcomitrella patens*) cells

**DOI:** 10.1186/1472-6750-5-30

**Published:** 2005-11-07

**Authors:** Andreas Schaaf, Stefanie Tintelnot, Armin Baur, Ralf Reski, Gilbert Gorr, Eva L Decker

**Affiliations:** 1 Department of Plant Biotechnology, Faculty of Biology, University of Freiburg, Schaenzlestr. 1, 79104 Freiburg, Germany; 2 greenovation Biotech GmbH, Boetzinger Str. 29b, 79111 Freiburg, Germany; 3 Department of Plant Biochemistry and Biotechnology, University of Münster, Hindenburgplatz 55, 48143 Münster, Germany

## Abstract

**Background:**

Efficient targeting to appropriate cell organelles is one of the bottlenecks for the production of recombinant proteins in plant systems. A common practice is to use the native secretory signal peptide of the heterologous protein to be produced. Though general features of secretion signals are conserved between plants and animals, the broad sequence variability among signal peptides suggests differing efficiency of signal peptide recognition.

**Results:**

Aiming to improve secretion in moss bioreactors, we quantitatively compared the efficiency of two human signal peptides and six signals from recently isolated moss (*Physcomitrella patens*) proteins. We therefore used fusions of the different signals to heterologous reporter sequences for transient transfection of moss cells and measured the extra- and intracellular accumulation of the recombinant proteins rhVEGF and GST, respectively. Our data demonstrates an up to fivefold higher secretion efficiency with endogenous moss signals compared to the two utilised human signal peptides.

**Conclusion:**

From the distribution of extra- and intracellular recombinant proteins, we suggest translational inhibition during the signal recognition particle-cycle (SRP-cycle) as the most probable of several possible explanations for the decreased extracellular accumulation with the human signals. In this work, we report on the supremacy of moss secretion signals over the utilised heterologous ones within the moss-bioreactor system. Though the molecular details of this effect remain to be elucidated, our results will contribute to the improvement of molecular farming systems.

## Background

The product range for recombinant biopharmaceuticals includes relatively simple proteins, for example insulin, and ends with candidates that require extensive secondary modification like erythropoietin or coagulation factor IX [1,2]. Commercial-scale production of proteins (molecular farming) nowadays is mainly performed with bacteria or mammalian cell-culture systems [1,3,4]. Bacterial production systems are highly efficient and well established for the production of comparably simple proteins but they come to their limits if complex posttranslational processing is required. One major problem is the absence of protein glycosylation in prokaryotes. Mammalian cell lines, on the other hand, perform human-like posttranslational modifications and offer a high product quality but hold risks of product contamination and high overall production costs (e.g. [5]).

Plants have emerged as a safe and cost-effective alternative to the traditional systems (e.g. [6,7]). They synthesize proteins with correct assemblage and the posttranslational modifications of higher eukaryotes. In contrast to microbial systems, glycosylated plant proteins carry complex-type glycans with the same core structure as human glycoproteins. The glycosylation pattern of plant proteins, however, differs from that in humans concerning two residues linked to the core structure as well as terminal sugar moieties [2,8]. The adaptation of the plant glycan pattern to its human counterpart (*humanisation*) poses a challenge for the implementation of plant systems with regard to the production of recombinant pharmaceutical proteins [9] as differences in the glycan structure could substantially alter protein characteristics and even act immunogenically in patients [10-13].

The moss *Physcomitrella patens *is the only known plant in which homologous recombination occurs in a frequency that allows its application as an engineering tool for targeted knock out and gene replacement. With this technique a humanisation of the moss glycosylation pattern was performed recently [14,15]. Together with photoautotrophic growth in bioreactors these advantages make *Physcomitrella *an ideal system for the production of plant-made pharmaceuticals [16].

In molecular farming approaches, recombinant proteins, which require correct posttranslational modifications, are often targeted to the secretory pathway. This, on the one hand, is due to the fact that enzymes responsible for various modifications, e. g. glycosylation and protein trimming, are located within the membranes of endoplasmic reticulum (ER) and Golgi apparatus. On the other hand, secretion of the protein of interest into the culture medium separates it from the pool of intracellular proteins and therefore drastically facilitates its purification and reduces costs for downstream processing [1].

A common way to achieve secretion of recombinant proteins is the utilisation of the native signal peptide, if any is present. The mechanism of protein secretion depends on the recognition of an N-terminal signal peptide and is conserved among all eukaryotes [17]. Though the general hydrophobic character of secretion signals is conserved between plants and animals, they are not universally interchangeable [18]. Additional features might contribute to the efficiency of signal peptide recognition [19]. In order to establish moss as a production system for recombinant proteins, we intended to enrich our toolboxes with a set of endogenous signal sequences from the moss. The availability of *Physcomitrella *secretion signals gives us the opportunity to secrete even proteins that are located within the cytosol in their host organism and thus lack a secretion signal.

Here, we describe the comparison of two heterologous signal peptides from human origin, the vascular endothelial growth factor (hVEGF) and the coagulation factor IX (hFIX), respectively, in the moss. Evaluation of a heterologous signal and the signal peptide of *Physcomitrella *aspartic proteinase PpAP1 [20] revealed better secretion of recombinant hVEGF (rhVEGF) with the moss signal.

In addition, the secretion efficiency of five putative signal peptides originating from recently isolated and identified *Physcomitrella *extracellular proteins xyloglucan endotransglycosylase/hydrolase (PpXTH1), pectin methylesterase (PpPME1), fasciclin-like protein (PpFLP), carbonic anhydrase-like protein (PpCALP), and lipid-transfer protein (PpLTP) (Tintelnot *et al*., manuscript in preparation) was tested in comparison to the PpAP1 secretion signal. These signal sequences were fused to hVEGF and amounts of secreted rhVEGF were quantified by ELISA measurements. All of the investigated moss signal peptides showed at least comparable or even higher secretion efficiency than the human signals. Besides, we underline the functionality of a transient production technique, that allows the fast production of recombinant proteins [21]. Within the field of molecular farming these techniques are essential for proof-of-concept studies prior to the establishment of production lines.

## Results

### Efficiency of human secretion signals in *Physcomitrella patens*

**Figure 1:**
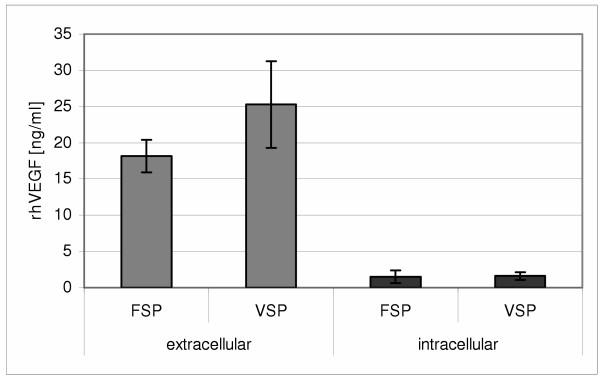
**Comparison of heterologous signals from human origin**. The secretion signals of human blood clotting factor IX (FSP) as well as human vascular endothelial growth factor (VSP) were fused to the coding sequence of vascular endothelial growth factor (hVEGF). Moss protoplasts were transiently transfected with the expression constructs and concentration of extracellular as well as intracellular rhVEGF was determined by ELISA. Mean values were taken from three transfections. Error bars indicate the absolute average deviation (AAD).

In order to improve transient production and secretion of pharmaceutically interesting proteins in the moss *Physcomitrella patens*, we first tested the efficiency of two human signal peptides. These were derived from the human blood clotting factor IX (hFIX) and the vascular endothelial growth factor (hVEGF). The secretion signals of both proteins, FSP and VSP, respectively, were tested with hVEGF as a reporter gene. *Physcomitrella *protoplasts were transiently transfected with the respective plasmids followed by quantification of extra- and intracellular concentrations of the recombinant proteins by ELISA (Fig. 1). Testing the hFIX signal peptide FSP, the moss cells secreted on average 18.17 ng/ml recombinant hVEGF (rhVEGF) to the culture supernatant, whereas the hVEGF signal VSP yielded a slightly higher average value (25.27 ng/ml). Comparison with intracellular concentrations of rhVEGF proved that both heterologous signals from human origin were quite effective in secreting the reporter protein from moss cells.

### Transient production of recombinant glutathione S-transferase

**Figure 2:**
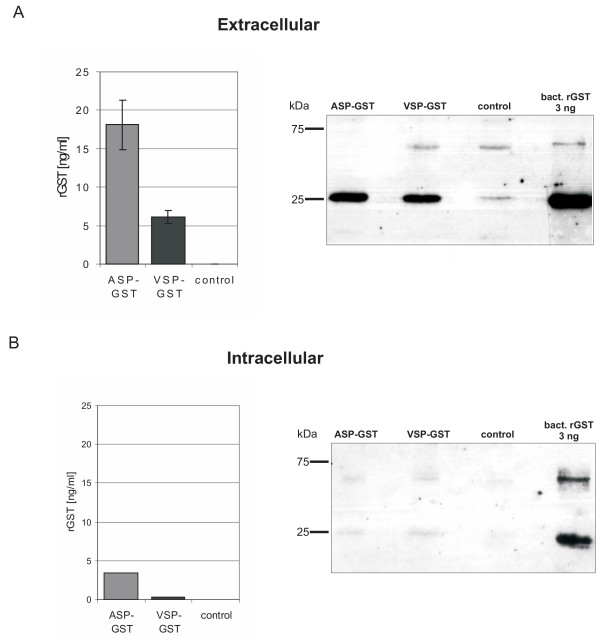
**Concentrations of secreted recombinant GST**. *Physcomitrella *protoplasts were transiently transfected with pVSP-GST and pASP-GST in eight independent transfections. After 5 days the concentrations of secreted and intracellularly-retained recombinant protein were measured by ELISA. Additionally, GST was affinity-purified from both medium and supernatant and detected by western blot. **A: **Culture medium of two transfections was pooled. Mean values of eight transfections are given. **B: **Protoplasts of eight transfections were pooled and GST was affinity-purified from the crude extracts. Values reflect the means of the two measurements. Control: mock-transfected protoplasts.

Having proven the general functionality of heterologous secretion signals we aimed to compare the efficiency of a human and a moss signal peptide. We chose VSP that secreted a slightly higher amount of the reporter protein, and ASP, a moss signal sequence of the recently characterised aspartic proteinase PpAP1 [20]. The signal sequences were fused to the cDNA of glutathione S-transferase (GST) from *Schistosoma japonicum*, which served as a reporter protein. Five days after transient transfection of moss cells, reporter concentrations were quantified by ELISA (Fig. 2). With the moss signal peptide ASP, the protoplasts secreted between 14.11 and 24.33 ng/ml rGST into the culture supernatant. On the contrary, the human signal peptide yielded values in the range from 4.50 to 7.17 ng/ml. Taken the means of these values, when using the moss signal peptide, the concentration of rGST in the medium (18.10 ng/ml) was almost three times higher than with the human signal (6.10 ng/ml) (Fig. 2A).

For the quantification of intracellular rGST, the crude protein extracts were directly measured by ELISA (Fig. 2B). The relation of the values correlated to the extracellular situation. ASP-GST-transfected protoplasts contained 3.38 ng/ml rGST whereas those transfected with VSP-GST reached a mean value of no more than 0.35 ng/ml.

For visualisation of recombinant GST on Western blots (Fig. 2), GST was affinity-purified by using GSH-Sepharose, and the eluted proteins were separated by non-reducing SDS-PAGE. GST was detected with an anti-GST antibody. The blots showed the main bands at the expected size of 26 kDa for the GST protein. The weak additional higher band was caused by dimer formation. Weak bands detected in the control lanes may derive from cross-reactivity of the anti-GST antibody to moss GST or be an artefact from the protein purification procedure.

### Transient production of recombinant human VEGF

**Figure 3:**
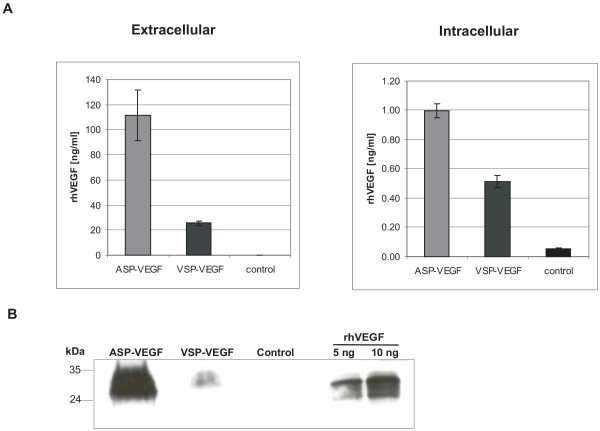
**Intra- and extracellular concentrations of recombinant rhVEGF**. *Physcomitrella *protoplasts were transiently transfected with pVSP-VEGF and pASP-VEGF in four (pVSP-VEGF and control) and seven (pASP-VEGF) independent transfections. After 5 days the concentrations of secreted and intracellularly retained recombinant protein were measured by ELISA. **A: **mean values of concentrations of extra- and intracellular rhVEGF measured by ELISA. **B: **Western blot with culture medium of transiently rhVEGF-producing cell. Control: untransfected moss protoplasts.

To examine whether the supremacy of the moss signal is specific for GST, we subsequently analysed the secretion efficiency of ASP and VSP with the hVEGF reporter protein. When the natural signal peptide was used, the amount of extracellular rhVEGF ranged between 21.90 and 29.50 ng/ml (mean of 5 transfections: 25.48 ng/ml; Fig. 3A). Using the moss signal peptide resulted in a more than fivefold increase of rhVEGF in the culture medium (mean of 7 transfections: 111.14 ng/ml; single values ranged from 75 ng/ml to 162 ng/ml). The differences in extracellular rhVEGF accumulation of ASP-VEGF vs. VSP-VEGF-transfected cells were also reflected in the Western analysis (Fig. 3B). Additionally, the amounts of intracellular rhVEGF were determined by ELISA. ASP-VEGF-transfected protoplasts contained 1.0 ng/ml rhVEGF. Those transfected with VSP-VEGF reached an average value of 0.51 ng/ml.

### Highly efficient secretion by endogenous signal peptides

**Figure 4:**
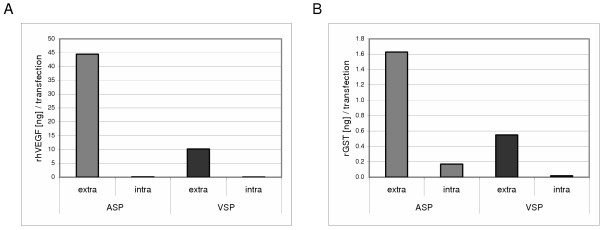
**Intra- and extracellular distribution of recombinant protein**. The relations between intra- and extracellular amounts of recombinant rhVEGF **(A) **and GST **(B) **are given. Values reflect the mean amounts of recombinant protein per transfection. **ASP: **plasmids with moss signal peptide; **VSP: **plasmids with human signal peptide; **extra: **amount of recombinant protein in culture medium; **intra: **intracellular amounts of recombinant protein.

Comparison of the intracellularly retained and extracellular proportions of rGST and rhVEGF, respectively, demonstrates that secretion from the moss cells was very efficient (Fig. 4). Most of rGST, i.e. 90.6%, was secreted to the culture medium. Up to 99.8% of the produced rhVEGF accumulated in the culture medium, whereas no significant amounts were found inside the cells, neither with the moss signal nor with the human one. Furthermore, the ratio of intra- to extracellular protein did not differ significantly between the human and the moss secretion signal. With respect to the biological processes, we suggest as the most probable among several possible reasons for the difference in secretion efficiency a less efficient translation process when using the human signal (see discussion).

**Figure 5:**
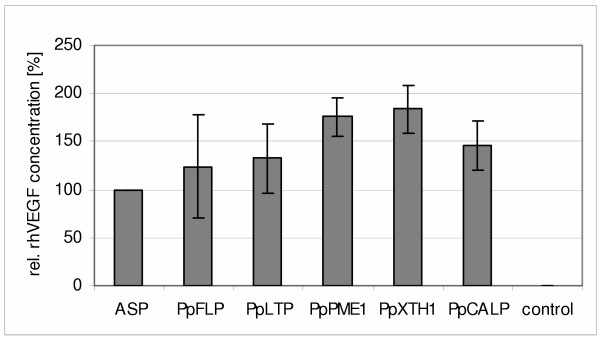
**Secretion efficiency of different moss signal peptides**. Signal sequences of PpAP1, PpFLP, PpLTP, PpPME1, PpXTH1, PpCALP were fused to rhVEGF. Shown are the mean values of three independent experiments with three transfections each time (n = 9). Prior to calculation of averages, outliers were removed if the z-score was >1. RhVEGF concentration of the PpAP1-construct was set to 100%. Error bars indicate the absolute average deviation (AAD) of the three experiments. Control: untransfected *Physcomitrella *protoplasts.

After having proven the high efficiency of one endogenous moss signal peptide in the moss system, we aimed to study the efficiency of additional moss signal sequences and tested five other putative secretion signals from *Physcomitrella *in transient transfection assays. These signal peptides originated from putative extracellular proteins isolated from moss culture medium (Tintelnot *et al*., manuscript in preparation). The signal sequences of PpFLP (fasciclin-like protein), PpLTP (lipid-transfer protein), PpPME1 (pectin methylesterase), PpXTH1 (xyloglucan endotransglycosylase/hydrolase), PpCALP (carbonic anhydrase-like protein) as well as PpAP1 were fused to rhVEGF in order to analyse their secretion efficiency by ELISA after transient transfection of moss protoplasts. All further investigated moss signal peptides showed comparable or even higher secretion efficiencies than the PpAP1 signal ASP (Fig. 5). The rhVEGF amount in the medium achieved by the signal peptides of PpPME1 and PpXTH1 was approximately 80% higher than with the PpAP1 signal.

**Figure 6:**
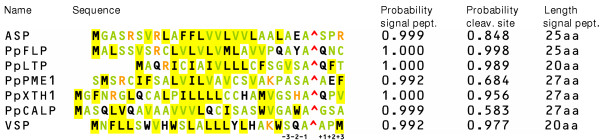
***Physcomitrella patens *signal peptides**. Amino acid sequences of signal peptides from PpAP1 (ASP), VEGF (VSP) and the extracellular proteins PpFLP, PpLTP, PpPME1, PpXTH1, PpCALP, and VEGF are shown as predicted by SignalP. Letters with yellow background represent hydrophobic amino acid residues (V, L, I, W, F, M), orange letters positive (K, R) and green letters small and neutral residues (A, C, G, N, P, S, T, V). The predicted cleavage sites are indicated as well as the positions adjacent to this site. In the right part of the figure probabilities for each signal peptide and the cleavage site given by the SignalP-HMM prediction as well as the length of the signal peptides are shown.

A comparison of the different moss signal peptides is shown in Fig. 6. According to SignalP, for the six cloned sequences the probabilities for being a signal peptide were always higher than 99% and their functionality *in vivo *was proven as demonstrated above. All of the signals comprise many hydrophobic amino acid residues in their central part indicating the hydrophobic region. The N-terminal part has at least one positive residue in most of the signals (except PpCALP and VSP, respectively), whereas the C-terminal part always contains a small and neutral residue at position -1 and -3 with respect to the cleavage site as it is proposed for signal sequences [22,23]. The probability for correct prediction of the cleavage site varied from 58.3% for PpCALP to 99.8% for PpFLP. High variations were also found regarding the overall length of the signal sequences, which ranges from 20 to 27 amino acids. Analysis of available *Physcomitrella *sequences resulted in an average length of 26 amino acids for 86 signal peptides predicted.

Taken together, the results of the transient production of recombinant proteins demonstrate that the secretion machinery of *Physcomitrella *works very efficiently. Furthermore it was shown that the utilisation of an endogenous signal peptide can lead to a remarkable increase in recombinant protein production in comparison with the heterologous sorting signals used in this study.

## Discussion

An important issue in molecular farming is the accumulation and downstream processing of recombinant proteins. Mostly, the proteins are targeted to the secretory pathway as the complete set of enzymes responsible for posttranslational modifications, especially for protein glycosylation, is located within the secretory compartments, i.e. ER and Golgi apparatus. In case of using suspension cultures as a production system secretion of proteins provides the additional advantage of easier protein purification.

Several systems involving secretion in protein production have been established. Among these are both, cell suspension cultures and whole plant systems, e.g. rhizosecretion [24,25] or production in the water plant *Lemna *[26]. Seed storage of the recombinant product also involves secretory targeting, as deposition in protein storage vacuoles is achieved by traffic via the secretory pathway [27].

A common practice for the production and secretion of human proteins in plants is to use their native signal peptide. This is possible because of the conservation of the signal recognition- and ER-insertion mechanisms throughout eukaryotes [17,28,29] and circumvents laborious steps of signal engineering. However, the amino-acid composition of signal peptides is extremely variable and the exact mechanism of their recognition by SRP and the translocon is not yet completely understood. Their determining feature is the hydrophobicity of a ten to fifteen residue core sequence, which surprisingly does not show any sequence conservation [30]. The functionality of the signals is only marginally impaired by amino-acid exchanges, as long as the hydrophobic core is not affected [22,31]. Exchanges within the core can in contrast totally impede signal recognition, even when leading to a higher hydrophobicity [32]. A closer look at the nature of the six moss signal peptides analysed in this study revealed a high percentage of the hydrophobic amino acids L, F, I, M, V and W [22] in the central part of the signal peptides, even though the hydrophobic stretches vary in length and position. N-terminal of the hydrophobic centre positively charged residues have been proposed [33]. Except for PpCALP, all of the moss signal sequence N-termini contain at least one arginine residue. For the signal peptide of PpCALP, which has no positively charged residue, the initiation methionine could perhaps compensate the lack of arginine [34]. All of the investigated signal sequences follow the (-3, -1)rule, which means that the two amino acids at position one and three previous to the cleavage site have to be small and neutral [23]. The HMM cleavage site prediction from SignalP showed a rather low probability for the signal sequences of PpPME1 (0.684) and of PpCALP (0.583). Additionally, in case of PpCALP, the SignalP-neural network predicted a signal peptide length of 32 amino acids instead of 27 amino acids which was predicted with SignalP-HMM and used in our study. However, experimental data showed that the sequences for both PpCALP and PpPME1 were not only functional, but resulted in a very efficient secretion of the rhVEGF protein. In addition, cleavage site prediction of ASP and VSP was verified experimentally. The analysis revealed that secretion dropped drastically when the last amino acid of a correctly predicted signal was deleted (data not shown).

The average length of eukaryotic signal peptides was calculated to be 22 amino acids [22,35], whereas the average length of 86 analysed *Physcomitrella *signal peptides is 26 residues. Although some correlations between the analysed *Physcomitrella *signal peptides and other eukaryotic secretion signals can be found, the variations within the amino acid composition remain high [34]. A functional role of additional, most likely structural determinants of signal peptides is assumed [19].

Previous studies evaluating endogenous vs. heterologous signal sequences in several species of the plant and animal kingdoms revealed for the majority a higher efficiency of kingdom-specific signals [18,36,37]. As secretion is one of the bottlenecks when establishing a production system, we aimed to define highly efficient signals to transport the recombinant proteins to the culture medium of the moss bioreactor system [16]. Thus we decided to compare six endogenous moss signal peptides plus two heterologous sequences from human origin. All of the moss signals turned out to drive secretion more efficiently than the human signal peptides. Two signals, the moss-derived ASP and the human VSP, were analysed in more detail with two different reporters, rGST (glutathione S-transferase) and rhVEGF (vascular endothelial growth factor), respectively. By secretion of the recombinant reporter protein GST we could show a threefold increase in extracellular accumulation with the ASP sequence compared to the human VSP. With the moss secretion signal, extracellular rhVEGF amount was up to fivefold more. Surprisingly, we did not find an increased intracellular accumulation in case of the human signal peptide. Amongst several possible alternatives (see below), our preferred explanation is that recognition by the SRP and maybe ER-insertion of the human signal peptide is suboptimal thus decreasing the translation rate of the recombinant protein. The differences in secretion efficiency clearly prove that besides hydrophobicity, some additional features, that may have a species-specific character, play a crucial role in signal recognition. As it is known that SRP occupies the EF-binding-site of the ribosome and thereby temporarily arrests translation [38], the cause for a reduced production rate with the human signal could be found in a hindered dissociation of SRP after docking of the translation complex to the translocon.

The second important interaction of the nascent signal peptide is performed with ER-membrane lipids as well as the translocon itself [39,40]. This interaction could demand unknown structural features of a signal peptide, which are not completely cross-host compatible, as mammalian and plant membranes differ significantly in their lipid composition [41].

However, besides a translational inhibition caused by hindered SRP-dissociation, alternative explanations for the worse performance of the human signals do exist: The protein in part may fail to be properly folded (perhaps because SP cleavage might not be efficient) and is subsequently degraded. Alternatively, transcription of that particular construct may be inefficient. The only possible determinant for such events can be found in the relatively short signal peptide sequences themselves, as all used vectors do not differ in any other detail. Apart from the above-mentioned translational inhibition, additional influence of the signal peptide on recombinant protein stability could only take place during, or shortly after ER-insertion. When the translation of the protein is completed, the signal peptides are cleaved. Thereafter, recombinant products do not differ in any residue and can therefore not exhibit different stabilities. Regulatory, post-targeting functions of cleaved signals are proposed by other authors and can therefore not be completely excluded in our case [42,43].

## Conclusion

Even if the structural background remains to be elucidated, our results demonstrate that the six moss sequences are more efficient than the two human ones used in our study in promoting secretion of recombinant human VEGF. In the interplay with optimised gene expression [44] and product stabilisation [45], the utilisation of moss-endogenous secretion signals could greatly enhance the productivity of the moss-bioreactor system.

## Methods

### Plant material and growth conditions

*Physcomitrella patens *(Hedw.) B.S.G. was grown axenically under standard conditions (agitated liquid modified Knop medium, 250 mg/l KH_2_PO_4_, 250 mg/l MgSO_4 _× 7H_2_O, 250 mg/l KCl, 1000 mg/l Ca(NO_3_)_2 _× 4H_2_O, 12.5 mg/l FeSO_4 _× 7H_2_O, pH 5.8) in a growth chamber or in photobioreactors (25 ± 1°C; light provided from outside by fluorescent tubes, Philips TL-D 36W/25; light flux of 55 μmol s^-1 ^m^-2^, light-dark regime of 16:8 h) as described [46].

### Prediction of signal sequences

Signal sequences of the putative extracellular *Physcomitrella *proteins Fasciclin-like protein (PpFLP [EMBL:AJ843251]), Lipid-transfer protein (PpLTP [EMBL:AJ843246]), Pectin methylesterase1 (PpPME1 [EMBL:AJ843245]), Xyloglucan endotransglycosylase/hydrolase1 (PpXTH1 [EMBLAJ843248]) and Carbonic anhydrase-like protein (PpCALP [EMBL:AJ843247]) (Tintelnot *et al*., manuscript in preparation) as well as for PpAP1 [20] were predicted by SignalP 3.0 Server [23,33,35].

For analysis of the average length of moss signal peptides, all 486 publicly available full-length *Physcomitrella *coding sequences as of May, 2005 were retrieved from Genbank and 86 putative signal peptides predicted using the SignalP 3.0 neural network. In addition, a set of 408 *Arabidopsis thaliana *coding sequences were retrieved using the query keywords "mature" and "peptide" and 39 putative signal peptides predicted.

### Construction of targeting vectors

For the construction of pASP-GST and pVSP-GST the coding sequence (cds) of GST was PCR-amplified with the primers GSTf (5'-AGATCTATGTCCCCTATACTAGGT-3') and GSTr (5'-GAGCTCTCACGGAACCAGATCCGATTT-3') with pGEX (Amersham Biosciences, Germany) serving as template. The PCR product was directly cloned into the pCR4-TOPO vector (Invitrogen, Germany). Introduced BglII and SalI restriction sites enabled the replacement of the GFP-cds of mAV4 [47] by the newly generated cds for GST. The resulting plasmid was named pGST. The ASP-signal was amplified with the primers ASPf (5'-AGATCTTGCCTCAGCTAAGGCTGC-3') and ASPr (5'-AGATCTTGCCTCAGCTAAGGC-3') using pSP-GFP [20] as template. The PCR product was directly cloned into pCR4-TOPO vector. For subcloning in pGST, the introduced SalI and BglII sites were used. The resulting plasmid was called pASP-GST.

For the construction of pVSP-GST, the plasmid pVSP-GFP served as a starting point. It is a derivative of mAV4, with the cds of the rhVEGF-signal sequence (VSP) fused upstream to GFP. The GST-cds was introduced by using the SalI and BglII restriction sites for replacing the GFP-cds.

The plasmids pVEGF and pASP-VEGF were constructed using the plasmids pRT101_C3_VEGF [48] containing the cds for human VEGF as starting point. The human VEGF signal was amplified from pRT101_P21 [48] with MOB_323 (5'-ATACTCGAGGAAGATGAACTTTCTGCTGTCTTGG-3') and MOB_349 (5'-CTGCCATGGGTGCAGCCTGGGACCAC-3') and cut at XhoI/NcoI restriction sites for ligation into pRT101_C3_VEGF. The ASP-signal from *Physcomitrella patens *(PpAP1) located on the plasmid pSP-GFP was amplified using MOB_642 (5'-GCCCTCGAGGAAGATGGGGGCATCGAGGAGT-3') and MOB_765 (5'-CTGCCATGGGTGCTGCCTCAGCTAAGGC-3'). By use of the restriction sites XhoI and NcoI the was cloned into pRT101_C3_VEGF.

Construction of the plasmid pUC-SP-VEGF was carried out by replacing the luciferase-cds and terminator in pluc-Actin [44] with the cds of rhVEGF and 35S-terminator from pAP1-SP-VEGF-His [15] via the enzymes Hind III and Nco I. The different secretion signals – plus four basepairs (GCAC) missing from the rhVEGF cds after Nco I digest – were cloned into the pUC-SP-VEGF via the restriction enzymes Nco I and Xho I after PCR amplification and direct cloning into the pCR4-TOPO vector (Invitrogen). The following primers were used for amplification: PME-SPf (5'-CGAGCGCAATGGGGAGCATGTC-3') and PME-SPr (5'-TCCATGGGTGCTGCTGACGCGGGCTTC-3'), XTH-SPf (5'-GCTGCAGAGAAATGGGGTTCAATAGAGG-3') and XTH-SPr (5'-TCCATGGGTGCAGCGTGACTGCCGAC-3'), CALP-SPf (5'-GCTCGAGGGCGATGGCGAGCCAACTTG-3') and CALP-SPr (5'-TCCATGGGTGCTGCCCACGCGCCAAC-3'), FLP-SPf (5'-GCTCGAGAACAATGGCGCTTTCGTCGG-3') and FLP-SPr (5'-TCCATGGGTGCGGCATACGCTTGAGGC-3'), GLP-SPf (5'-GCTCGAGTAACATGGCTGCCCGTTTCG-3') and GLP-SPr (5'-TCCATGGGTGCAGCATACACCATGGCC-3'), LTP-SPf (5'-GCTCGAGGAGGATGGCACAACGCATTTG-3') and LTP-SPr (5'-TCCATGGGTGCAGCAGACACTCCAGAG-3'), and AP-SPf (5'-TCTCGAGGACGATGGGGGCATCGAGGAG-3') and AP-SPr (5'-TCCATGGGTGCTGCCTCAGCTAAGGCTG-3') for the endogenous signal peptides as well as VEGF-SPfor (5'-TGCTCGAGCGAGATGAACTTTCTGCTGTC-3') and rev (5'-TTCCATGGGTGCAGCCTGGGACCACTTG-3') for the heterologous secretion signal.

### Transfection of *Physcomitrella *protoplasts

The DNA for transfection was prepared with Qiagen's Plasmid Maxi kit. A total number of 3 × 10^5 ^protoplasts was transiently transfected with 30–50 μg of circular plasmid DNA. Protoplasts were isolated from bioreactor-grown material and transfected as described [46]. After transfection, the protoplasts were suspended in 100 μl 3 M-medium supplemented with 0.01% BSA and subsequently transferred to 96 well plates (Nunclon™ surface; Nunc, Denmark). After 24 hrs the culture medium was replaced with fresh medium. Five days after transfection the supernatant was harvested. Protoplasts were resuspended in 100 μl PBS buffer supplemented with 1% Triton X100 and 1% plant protease inhibitor cocktail (Sigma, Germany). These samples were immediately processed with protein extraction.

### Extraction of intracellular protein

For the extraction of intracellular protein, glass beads (1 mm diameter) were added to the tubes. Cell disruption was carried out in a beadmill (Tissuelyzer, Qiagen) for 1 min at 30 Hz. Cell debris was sedimented (14,000 rpm, 10 min, 4°C) and the supernatant was transferred into a fresh tube for further assaying.

### Measurement of protein concentration by ELISA

Extra- and intracellular concentrations of recombinant protein were determined by ELISA. In order to adjust pH and ionic conditions, the harvested culture medium was supplemented with an equal volume of 2 × PBS and kept on ice until ELISA measurement or stored at -80°C. Cell extracts were directly applied to the ELISA. All samples were diluted appropriately and measured in duplicates. Separate standard dilutions were prepared for medium- and cell extract-conditions respectively, because of the samples' different buffer conditions. For determination of recombinant GST concentrations, the 96 well detection module (Amersham Biosciences) was used following the manufacturer's instructions. Recombinant hVEGF samples were analysed by sandwich ELISA (anti-hVEGF, capture: #AF-293-NA, anti-hVEGF, detection: #BAF-293, rhVEGF_121_, standard: #298-VS, R&D, Germany; Nunc-Immunosorb plates). Dilutions were made in 1 × PBS/0.1% BSA (Serva, Germany).

### Purification of GST

Recombinant GST was affinity-purified using GSH-Sepharose (Gluthathione Sepharose 4B, Amersham Biosciences). Therefore a 50%-slurry of the GSH-Sepharose was prepared according to the manufacturer's instructions and 1/50 volume of the slurry was added to the protein solution. In case of extracellular GST, 1/10 volume of 10 × PBS buffer was added to adjust pH of the culture medium. Binding was carried out for 2 hours at room temperature with gentle end-over-end rotation. GSH-Sepharose was sedimented at 500 × g for five minutes and the supernatant was discarded. The sepharose was washed once with ten bed volumes of PBS buffer. After adding 50 μl of SDS sample buffer, the samples were heated at 95°C for five minutes, and centrifuged for one minute at 13,000 × g. The resulting supernatant was analysed by SDS-PAGE followed by Western blot.

### SDS-PAGE and western blot

For the visualisation by Western blot, the affinity-purified GST was first separated on a 12% SDS gel following standard techniques. The separated proteins were then transferred to a PVDF membrane (Immobilon-P, Millipore, Germany) with the semi-dry method. Transfer was carried out according to the instructions given by the membranes manufacturer. For signal detection, the ECL advance western blotting detection kit (Amersham Biosciences) was used. All steps were carried out according to the manufacturer's protocol. Anti-GST antibody (developed in rabbit, Sigma) was used at a 1:5,000 dilution. Anti-rabbit antibody (peroxidase-coupled, developed in donkey, Amersham Biosciences) was used for detection in a 1:500,000 dilution. For rhVEGF, anti-human VEGF (#AF-293-NA, 1:500), anti-goat IgG (#A5420, Sigma, 1:8000) and rhVEGF as standard was used.

## Authors' contributions

AS carried out cloning, ELISA and western blot analyses of the GST-fusion constructs and drafted the main part of the manuscript. ST performed cloning and ELISA studies as well as computational analysis of the *Physcomitrella *signal peptides and was involved in preparing the manuscript. AB carried out cloning, ELISA and western blot analyses of the VEGF-fusion constructs. RR leads the chair plant biotechnology at Freiburg University. GG is scientific director of greenovation Biotech GmbH and guided the work on ASP-VEGF and VSP-VEGF. ELD was responsible for the design and development of these studies and writing of the manuscript; consequently she is the corresponding author. All authors read and approved the final manuscript.
